# Identification of KASP markers and candidate genes for drought tolerance in wheat using 90K SNP array genotyping of near-isogenic lines targeting a 4BS quantitative trait locus

**DOI:** 10.1007/s00122-023-04438-3

**Published:** 2023-08-16

**Authors:** Guannan Liu, Dongcheng Liu, Aimin Zhang, Hui Liu, Md Sultan Mia, Daniel Mullan, Guijun Yan

**Affiliations:** 1 UWA School of Agriculture and Environment and The UWA Institute of Agriculture, The University of Western Australia, Perth, WA 6009 Australia; 2 State Key Laboratory of North China Crop Improvement and Regulation, College of Agronomy, Hebei Agriculture University, Baoding, 071000 Hebei China; 3 InterGrain Pty. Ltd., 19 Ambitious Link, Bibra Lake, WA 6163 Australia

## Abstract

**Key message:**

This study identified a novel SNP and developed a highly efficient KASP marker for drought tolerance in wheat by genotyping NILs targeting a major QTL for drought tolerance using an SNP array and validation with commercial varieties.

**Abstract:**

Common wheat (*Triticum aestivum* L.) is an important winter crop worldwide and a typical allopolyploid with a large and complex genome. With global warming, the environmental volatility and incidence of drought in wheat-producing areas will increase. Molecular markers for drought tolerance are urgently needed to enhance drought tolerance breeding. Here, we genotyped four near-isogenic line (NIL) pairs targeting a major QTL *qDSI.4B.1* on wheat chromosome arm 4BS for drought tolerance using the 90K SNP Illumina iSelect array and discovered a single nucleotide polymorphism (SNP) (Excalibur_c100336_106) with consistent genotype–phenotype associations among all four NIL pairs and their parents. Then, we converted the SNP into a Kompetitive Allele-Specific PCR (KASP) marker, with an accuracy of 100% for the four NIL pairs and their parents and as high as 81.8% for the 44 tested wheat lines with known phenotypes collected from Australia and China. Two genes near this SNP were suggested as candidate genes for drought tolerance in wheat after checking the Chinese Spring reference genome annotation version 1.1. One gene, *TraesCS4B02G085300,* encodes an F-box protein reportedly related to the ABA network, a main pathway for drought tolerance, and another gene, *TraesCS4B02G085400,* encodes a calcineurin-like metallophos-phoesterase transmembrane protein, which participates in Ca^2+^-dependent phosphorylation regulatory system. Based on this work and previous research on pre-harvest sprouting, we established a quick and efficient general SQV-based approach for KASP marker development, integrating genotyping by SNP arrays (S) using NILs targeting major QTL for a specific trait (Q) and validating them with commercial varieties (V). The identified SNP and developed KASP marker could be applied to marker-assisted selection in drought breeding, and further study of the candidate genes may improve our understanding of drought tolerance in wheat.

**Supplementary Information:**

The online version contains supplementary material available at 10.1007/s00122-023-04438-3.

## Introduction

Common wheat (*Triticum aestivum* L.) is one of the most important cereal crops and a staple food grain for approximately 35% of the world’s population (Grote et al. 2021). Wheat is widely planted in the temperate zones north to a latitude of 67°and south to a latitude of 45° (King et al. 2018; Olmstead and Rhode 2011). However, abiotic stress significantly affects wheat quality and yield. As global temperatures have risen in recent years, drought has become a major abiotic stress for wheat, especially in rain-fed or limited irrigation environments (Akpınar et al. 2013; Hikmet et al. 2013). Unfortunately, the unavoidable causes of drought include global warming, underground water depletion, and erratic rainfall patterns, leading to water scarcity in agroecosystems worldwide, particularly in semi-arid, sub-tropical, and tropical drylands. Therefore, an effective response to drought effects is urgently needed in modern wheat breeding. The most promising and economically viable solution is using new cultivars with high drought tolerance.

Drought tolerance is a complex trait with several physiological and biochemical processes at the cellular and organism level and different stages of plant development (Ferdous et al. 2015; Langridge and Reynolds 2015; Lippmann et al. 2019). Plants adapt to drought stress in various ways, for example, by enhancing water uptake through deep root systems, reducing water loss by increasing stomatal sensitivity, and accumulating cellular osmolytes (Li et al. 2022). The complexity of drought tolerance has hindered the development of drought-tolerant varieties using only a single breeding approach. Hence, modern molecular breeding approaches can be developed to complement other methods to produce new cultivars with high tolerance to drought. In view of this, finding more stable and effective markers and genes conferring drought tolerance in wheat is crucial.

Compared to traditional marker-assisted selection, which is well recognized for its gap between expected and actual impacts, high-density single nucleotide polymorphism (SNP) genotyping arrays provide a much more efficient tool for discovering molecular markers. In the SNP genotyping, the 90K gene-associated SNP chip array (90k Wheat Illumina Infinium array) has been used to characterize genetic variation in allohexaploid wheat populations (Avni et al. 2014; Wang et al. 2014) for its excellent value in terms of genome-coverage. In addition, the recently created Kompetitive Allele-Specific PCR (KASP) markers have been used in marker-assisted plant breeding in pre-harvest sprouting, stripe rust, and powdery mildew resistance due to their cost-effectiveness and high throughput (Han et al. 2020; Liu et al. 2015; Makhoul et al. 2020). However, very few KASP markers have been developed for drought tolerance, and none have been validated in commercial cultivars. Therefore, it is vital to develop efficient and reliable KASP markers for drought tolerance in wheat using the 90K gene-associated SNP chip.

Besides the effective screening methodology, the NIL materials used in this study are also important which could reduce the genetic background noise in marker analysis. In earlier studies, recombinant inbred lines have been mainly used for molecular marker development for drought tolerance, which can require significant lengths of time for researchers to identify the target loci from all the 21 wheat chromosomes. Near-isogenic lines (NILs) could be used as a more valuable resource for studying specific genome positions as they transform complicated quantitative traits into a Mendelian factor (qualitative trait). Our laboratory recently developed a set of NILs targeting drought tolerance on chromosome arm 4BS and reported that the presence of the drought tolerance alleles increased drought tolerance by up to 14% across the NILs (Mia et al. 2019). Therefore, it will be valuable to use these NILs to develop molecular markers and identify genes closely related to this source of drought tolerance in wheat.

Taking advantage of combining NILs targeting drought tolerance, the 90K SNP chip assay, and the Chinese Spring wheat genome sequence data (Appels et al. 2018), this research developed a highly efficient and reliable KASP marker to assist drought tolerance breeding in wheat and identifies two putative genes for drought tolerance in wheat, which may provide a valuable reference for future drought regulation research.

## Materials and methods

### Plant materials and trait phenotyping

Four NIL pairs significantly differing in drought tolerance—developed by our laboratory from a cross of C306× Dharwar Dry (C306 is the donor parent of the tolerant allele of the QTL) using the heterogeneous inbred family (HIF) method coupled with immature embryo culture-based fast generation-cycling system with 8 generation of selfing. (Mia et al. 2019; Yan et al. 2017; Zheng et al. 2013)—were used as plant material. The genetic background of the isolines in each NIL pair, namely qDSI.4B.1-2, qDSI.4B.1-3, qDSI.4B.1-6, and qDSI.4B.1-8 (Mia et al. 2019), is almost identical, except at the target 4BS QTL, *QDSI.4B.1*, a major QTL explaining up to 14% of the phenotypic variation for drought tolerance (Kadam et al. 2012). The phenotypes of the NILs were previously tested in a post-anthesis water stress experiment in a temperature-controlled and naturally lit glasshouse (Mia et al. 2017).

Thirty-four newly released or historically domesticated commercial cultivars with known drought response, collected from Australia and China, were used for KASP marker validation. Their phenotypic data for drought response were obtained from previous studies by Grains Research and Development Corporation (GRDC), Australia (McDonald 2016; Shackley et al. 2019; Shackley et al. 2020; Shackley et al. 2021; State of Victoria 2018; GRDC 2019; Brown and Harris 2019) and Hebei Agricultural University (HAU), China (Tables S1, S2 and S3) based on their phenotypic data for drought response related traits (drought index,growing geography, and cultivar character descriptions). Because each state and country has its own ways of showing drought response level, we unified them into one standard, i.e., T for tolerant (drought index > 0.04 or grown in arid/semi-arid zone), S for sensitive (drought index ≤ 0.01 or grown in mid-high rainfall zone). For Australian cultivars, the drought response phenotype was collected from 2013 to 2022 in five states based on Australia’s crop sowing guide handbooks (Western Australia, Queensland, South Australia, New South Wales, and Victoria). The cultivars reported across at least two states and/or at least two years were chosen for this study. For Chinese cultivars, their drought tolerance phenotypes were collected from their National or Provincial seed ID descriptions based on the standard of technical specification of identification and evaluation for drought resistance in wheat (GB/T 21127-2007). Of the 34 cultivars, 16 are sensitive to drought, and 18 are tolerant (Table 1).

**Table 1:** Comparison of KASP marker and drought phenotype in 44 cultivars/lines

Cultivars/lines	Phenotype	Country	DE-106	Cultivars/ lines	Phenotype	Country	DE-106
C306 (♀)	T	Australia	** + **	LRPB Spitfire	T	Australia	−
Dharwar Dry (♂)	S	Australia	−	Sunvale	T	Australia	+
qDSI.4B.1-2-S	S	Australia	−	Clearfield JNZ	T	Australia	+
qDSI.4B.1-3-S	S	Australia	−	Condo	T	Australia	+
qDSI.4B.1-6-S	S	Australia	−	AGT Katana	T	Australia	+
qDSI.4B.1-8-S	S	Australia	−	Drysdale	T	Australia	−
qDSI.4B.1-2-R	T	Australia	+	EGA Wedgetail	S	Australia	+
qDSI.4B.1-3-R	T	Australia	+	LRPB Merlin	S	Australia	−
qDSI.4B.1-6-R	T	Australia	+	SQP Revenue	S	Australia	−
qDSI.4B.1-8-R	T	Australia	+	Sunstate	S	Australia	−
Malan1	T	China	+	Impose CL Plus	S	Australia	−
Longmai671	T	China	+	Kennedy	S	Australia	−
Longmai479	T	China	+	Pugsley	S	Australia	−
Shiai7	T	China	+	Fortune	S	Australia	−
Shimai22	T	China	+	EGA Eagle Rock	S	Australia	−
Shimai15	T	China	+	Chara	S	Australia	−
Hengguan35	T	China	−	DS Darwin	S	Australia	−
Henong825	T	China	+	LRPB Gauntlet	S	Australia	−
LRPB Impala	T	Australia	+	Forrest	S	Australia	−
Cosmick	T	Australia	+	Wallup	S	Australia	−
Hunter	T	Australia	−	LRPB Trojan	S	Australia	+
Axe	T	Australia	−	Tammarin Rock	S	Australia	+

*T* Tolerant phenotype, *S* Susceptible phenotype, + Tolerant allele, and − Susceptible allele

### DNA extraction and 90K SNP genotyping

Two-week-old young leaves were collected for genomic DNA extraction. The protocol for DNA extraction followed the CTAB method (Murray and Thompson 1980). Genotyping of the four NIL pairs and their parents (Dharwar Dry and C306) was carried out using the 90K SNP Illumina iSelect array. SNP clustering and genotype call steps were determined using GenomeStudio 2.0 software (Illumina) (Wang et al. 2018a). SNPs with a call frequency < 0.8 (i.e., missing data points > 20% or SNPs with no polymorphism) or minor allele frequency (MAF) < 0.05, and SNPs with > 0.25 heterozygous calls, were removed to obtain the raw SNP data.

### Screening SNP markers for drought tolerance

The raw SNP data were filtered to generate the genotypic data according to the reference SNP chromosome position (Mayer et al. 2014; Wang et al. 2014). The filtered SNPs were classified into two classes (tolerant or susceptible) based on the corresponding phenotype data. The SNPs on chromosome arm 4BS with > 50% genotype–phenotype associations among the four NIL pairs were selected for further study.

### KASP marker development and validation

The selected SNPs with polymorphism in or near the target QTL (*QDSI.4B.1*) for drought tolerance on chromosome arm 4BS were converted into KASP markers for genotyping in the validation population. KASP primers were designed using PolyMarker (http://www.polymarker.info/designed_primers) by selecting the standard primers in the Wheat iSelect 90 k section. The junction sequence of the fluorescent moiety FAM (6-carboxy-fluorescein) and HEX (Hexachlorofluorescein) was added to each of the two KASP 5’ ends of forward primers for genotyping. Protocols for the KASP marker preparation and PCR conditions are given in the KASP manual (https://www.biosearchtech.com/support/education/kasp-genotyping-reagents/running-kasp-genotyping-reactions) using SNPline™ instrumentation. The PCR was performed in a total volume of 3 μl using a 384-well plate format. The KASP genotyping assays were screened and read by Kraken™ software (v16.3.16.16288). The KASP primer mix, KASP 2× Mastermix, and 384-well plates were purchased from LGC Science Shanghai Ltd. (LGC). The KASP-related experiments were performed on the SNPline™ platform by LGC. The KASP marker was validated by testing the four NIL pairs and their two parents, and then, 34 commercial cultivars were collected from Australia and China with phenotype data for drought tolerance, obtained from published papers and the Grains Research and Development Corporation (GRDC).

### Identification and annotation of candidate genes

The SNPs on chromosome arm 4BS that could generate high-efficiency KASP markers (over 75% accuracy rate) were chosen to identify candidate genes for drought resistance. The genes containing or flanking these SNPs were subject to a BLASTN (Basic search) on the IWGSC Chinese Spring genome database (https://urgi.versailles.inra.fr/jbrowseiwgsc/gmod_jbrowse) using IWGSC Annotation v1.1. The candidate gene functions were searched in the online GrainGenes database (https://wheat.pw.usda.gov/GG3/), PlantGDB (http://www.plantgdb.org/), NCBI (https://www.ncbi.nlm.nih.gov/), EnsemblPlants (http://plants.ensembl.org/), Uniprot (https://www.uniprot.org/), IWGSC Annotation v1.0 reference to obtain more annotation information, including domain, gene family, structure, and biological functions.

### *In silico* expression of candidate genes

The selected candidate genes were further screened in WheatOmics (http://wheatomics.sdau.edu.cn/) to reveal gene expression in the Hexaploid Wheat Expression Database (IWGSC Annotation v1.1) under drought stress in their gene-expression databases (Ma et al. 2021).

### Statistical analysis

Student’s *t*-test was used to perform significance analysis in Excel 2013.

## Results

### Screening of candidate SNPs for drought tolerance

81,587 SNPs were identified using the 90K iSelect genotyping array and four NIL pairs targeting the drought tolerance locus on chromosome arm 4BS created from a cross of C306 and Dharwar Dry. Of which, 31,821 were called across the 21 wheat chromosomes after removing the SNPs that did not show polymorphism or did not satisfy the selection criteria, with 7157 SNPs assigned to the targeted chromosome 4B. Six SNPs had genotype–phenotype associations between tolerant and susceptible isolines of at least two of the four NIL pairs for drought tolerance. Of the six SNPs, Excalibur_c100336_106 (in bold in Table 2) had 100% genotype–phenotype matching among all four NIL pairs (Table 2).

**Table 2:** Genotype calling of the SNPs showing associations with phenotypic differences between resistant and susceptible isolines in at least two of the four NIL pairs

SNP	NIL2(T)	NIL3(T)	NIL6(T)	NIL8(T)	NIL2(S)	NIL3(S)	NIL6(S)	NIL8(S)	C306	Dharwar Dry
***Excalibur_c100336_106***	**BB**	**BB**	**BB**	**BB**	**AA**	**AA**	**AA**	**AA**	**BB**	**AA**
Excalibur_c39876_403	AA	AA	AA	AA	BB	BB	AA	AA	AA	BB
IAAV5564	AA	AA	AA	AA	BB	BB	AA	AA	AA	BB
Kukri_c64195_1044	BB	BB	AA	BB	AA	AA	AA	BB	BB	AA
Kukri_c64195_346	BB	BB	AA	AA	AA	AA	AA	AA	BB	AA
Kukri_c64195_432	AA	AA	BB	BB	BB	BB	BB	BB	AA	BB

*T* Drought tolerant, *S* Drought susceptible. NIL2(T), NIL2(S), NIL3(T), NIL3(S), NIL6(T), NIL6(S), NIL8(T), and NIL8(S) correspond to the four NIL pairs (qDSI.4B.1-2-R and qDSI.4B.1-2-S, qDSI.4B.1-3-R and qDSI.4B.1-S, qDSI.4B.1-6-R and qDSI.4B.1-6-S, qDSI.4B.1-8-R and qDSI.4B.1-8-S) generated from a C306× Dharwar Dry cross targeting *QDSI.4B.1*, a major QTL explaining up to 14% of the phenotypic variation for drought tolerance. The bold and italic marked SNPs indicate those with different genotypes in all four pairs between resistant and susceptible isolines

**Fig. 1:**
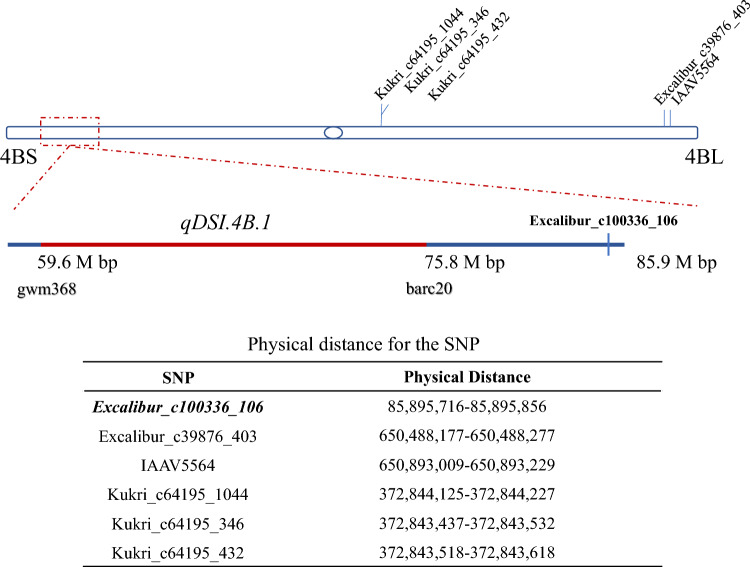
Locations of the six selected SNPs with their physical positions on chromosome 4B. SNP marked as bold shows genotype–phenotype associations in all four NIL pairs; red line is the peak region of the target QTL (*qDSI.4B.1*); QTL markers are bolded; the details of each SNP’s physical distance (based on Chinese Spring genome version 1.1) were listed in the embedded table

Based on our similar study on pre-harvest sprouting, we found that the SNPs with perfect genotype–phenotype associations in at least two NIL pairs could be useful for marker selection (Liu et al. 2022). Therefore, the above-mentioned six SNPs were all chosen for further analysis. Their sequences were subjected to BLASTN searches on the IWGSC reference genome sequence (V1.1) and GrainGenes databases to identify whether they flank or fall within the target QTL (*QDSI.4B.1*) on chromosome arm 4BS (Fig. 1). Five of the SNPs (Excalibur_c39876_403, IAAV5564, Kukri_c64195_1044, Kukri_c64195_346, and Kukri_c64195_432) are on the 4B long arm, with the other (Excalibur_c100336_106) located on 4BS near the target QTL. This SNP has 100% genotype–phenotype association in all four NIL pairs and is close to the peak of the target QTL (*QDSI.4B.1*). Therefore, this SNP was selected for further analysis.

### Conversion of the selected SNP into KASP marker and its efficiency validation

**Fig. 2:**
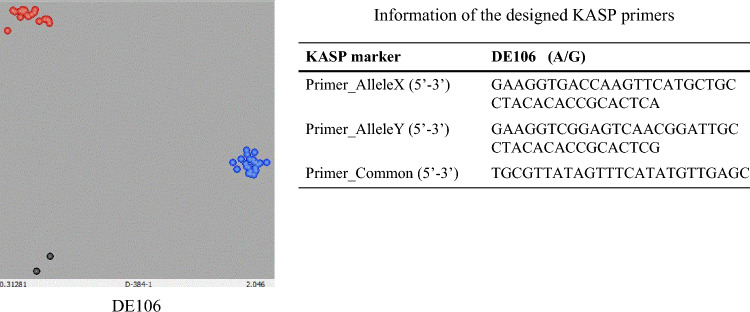
Genotyping plot of KASP assays for the KASP marker DE106 on 44 wheat cultivars/lines with different drought resistance performance (left part) and information on the KASP primers (right part) designed based on the sequence of the qualified SNP Excalibur_c100336_106. Note: X-axis: Allele 1, reported by FAM-type fluorescence; Y-axis, Allele 2, reported by HEX-type fluorescence; Blue dots are homozygous allele group 1 (resistance genotype allele BB); Red dots are homozygous allele group 2 (susceptible genotype allele AA); Black dots are blank controls

The selected SNP, Excalibur_c100336_106, was converted into a KASP marker, designated DE106 (Fig. 2). The KASP marker was used to check all four NIL pairs and their parents, which separated perfectly in the KASP assay (Fig. 2) and expressed the same results as the phenotype screening (Table 1). Therefore, the KASP marker DE106 had 100% accuracy for the drought response phenotype in the four NIL pairs and their two parents and was reliable and worthy of further validation.

Thirty commercial wheat cultivars (Table 1) with different drought responses were used to validate the efficiency of the KASP marker DE106. The results are in Table 1 and Fig. 2.

The 44 genotypes, including the four NIL pairs and their two parents, were separated into two clusters (Fig. 2): allele group 1 (blue dots are susceptible alleles) and allele group 2 (red dots are resistant alleles). The KASP marker had 81.8% conformity between genotype and phenotype (Tables 2 and 3).

**Table 3:** Accuracy of KASP marker DE106 in 44 cultivars/lines, including four NIL pairs

Genotype (Number)	Accuracy (%)	*P*-value
NILs (8)	100	–
NILs and cultivars (44)	81.8	2.06E−05**
Cultivars (36)	77.8	1.70E−03**

**P-value significant at the 0.01 level

### Candidate genes for drought tolerance on chromosome arm 4BS

**Fig. 3:**
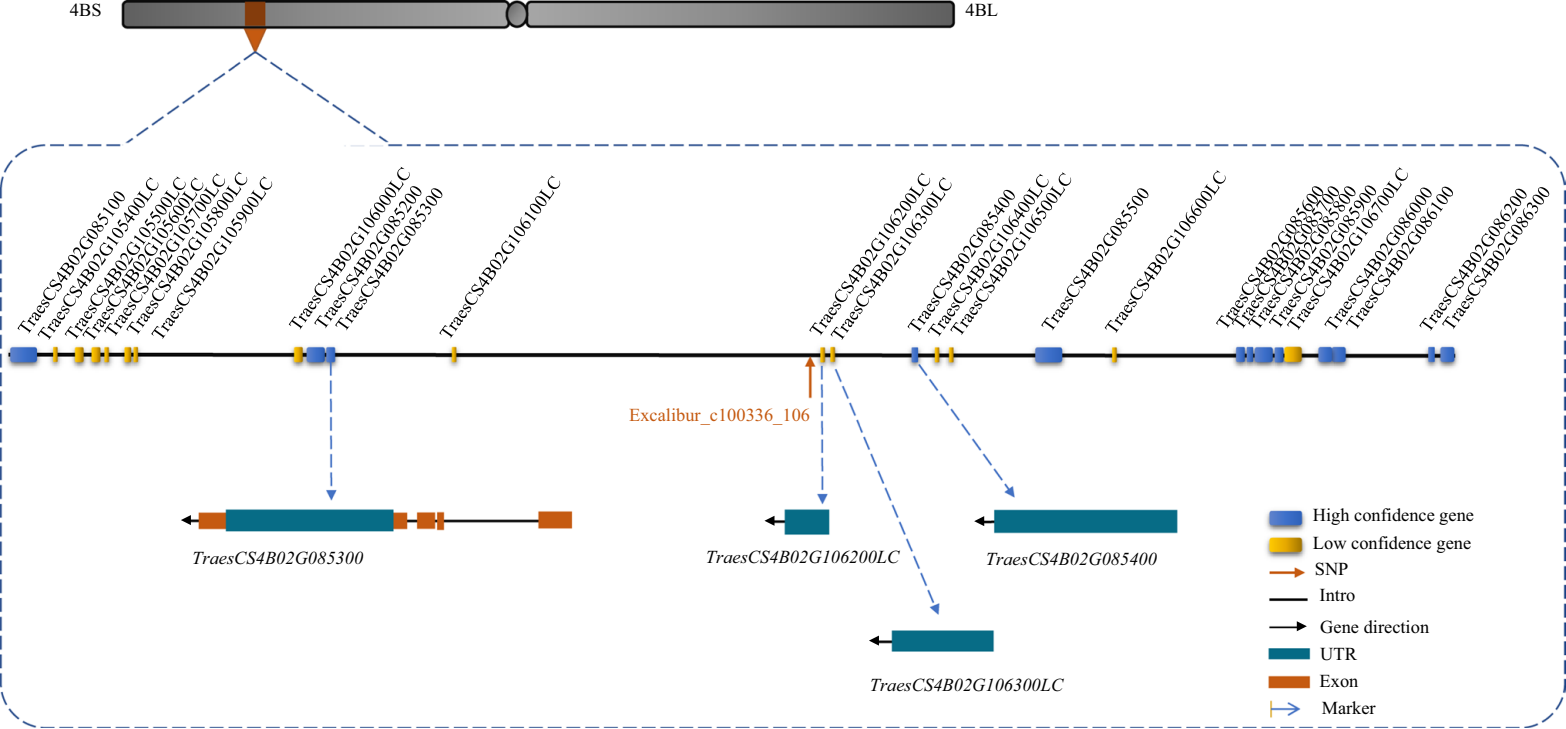
Genes within 1 Mbp from SNP Excalibur_c100336_106

The SNP, Excalibur_c100336_106, used to generate a high-efficiency KASP marker for drought tolerance evaluation, was subsequently used to search candidate genes for drought tolerance in the chromosome arm 4BS of the Chinese Spring reference wheat genome (version 1.1, accessed 5 November, 2022). Thirteen high-confidence and 14 low-confidence genes were detected within 1 Mbp distance from the SNP. Three high-confidence genes and eight low-confidence genes were upstream of the candidate SNP, and ten high-confidence genes and six low-confidence genes were downstream (Fig. 3). Detailed information on the 27 genes is in Table 4. Downstream, *TraesCS4B02G085400* is the closest high-confidence gene to the SNP, with a distance of 41,468 bp, while *TraesCS4B02G106200LC* is the closest low-confidence gene to the SNP, with a distance of only 570 bp.

**Table 4:** Genes related to drought tolerance in wheat and their function

No	Gene		Gene start	Gene end	Gene annotation
1	*TraesCS4B02G085100*	HC	85,581,887	85,586,551	Transmembrane protein, putative
2	*TraesCS4B02G105400LC*	LC	85,598,902	85,599,124	RNA-directed DNA polymerase (reverse transcriptase)-related family protein
3	*TraesCS4B02G105500LC*	LC	85,608,792	85,609,328	D-3-phosphoglycerate dehydrogenase
4	*TraesCS4B02G105600LC*	LC	85,613,355	85,613,582	Retrotransposon protein, putative, LINE subclass
5	*TraesCS4B02G105700LC*	LC	85,616,886	85,617,506	Retrotransposon protein, putative, unclassified
6	*TraesCS4B02G105800LC*	LC	85,631,483	85,632,322	Endonuclease/exonuclease/phosphatase family protein
7	*TraesCS4B02G105900LC*	LC	85,633,033	85,633,512	Ta11-like non-LTR retrotransposon
8	*TraesCS4B02G106000LC*	LC	85,694,622	85,695,505	Peptidase S24/S26A/S26B/S26C family protein
9	*TraesCS4B02G085200*	HC	85,695,748	85,701,883	Plant regulator RWP-RK family protein
10	*TraesCS4B02G085300*	HC	85,702,285	85,706,056	F-box/LRR protein
11	*TraesCS4B02G106100LC*	LC	85,754,476	85,754,832	Retrotransposon protein, putative, Ty3-gypsy subclass
12	***TraesCS4B02G106200LC***	**LC**	**85,896,356**	**85,896,694**	Calcineurin-like metallophosphoesterase super-family protein
13	*TraesCS4B02G106300LC*	LC	85,898,558	85,899,046	phytochelatin synthase 2
14	***TraesCS4B02G085400***	**HC**	**85,937,254**	**85,938,821**	Calcineurin-like metallophosphoesterase super-family protein
15	*TraesCS4B02G106400LC*	LC	85,941,475	85,942,068	Retrotransposon protein, putative, unclassified
16	*TraesCS4B02G106500LC*	LC	85,945,516	85,946,310	Endonuclease/exonuclease/phosphatase family protein
17	*TraesCS4B02G085500*	HC	85,981,861	85,987,870	Subtilisin-like protease
18	*TraesCS4B02G106600LC*	LC	86,008,089	86,009,252	Glutathione S-transferase T3
19	*TraesCS4B02G085600*	HC	86,058,611	86,061,451	RNA polymerase sigma factor RpoD
20	*TraesCS4B02G085700*	HC	86,061,679	86,063,684	Pentatricopeptide repeat-containing protein
21	*TraesCS4B02G085800*	HC	86,064,930	86,072,678	Protein transport protein SEC23
22	*TraesCS4B02G106700LC*	LC	86,072,682	86,080,334	Cytochrome P450
23	*TraesCS4B02G085900*	HC	86,074,788	86,078,669	Acetolactate synthase small subunit
24	*TraesCS4B02G086000*	HC	86,082,416	86,084,323	transmembrane protein, putative (DUF594)
25	*TraesCS4B02G086100*	HC	86,091,902	86,094,690	NADP-dependent alkenal double bond reductase
26	*TraesCS4B02G086200*	HC	86,130,835	86,132,031	Kinase-like
27	*TraesCS4B02G086300*	HC	86,139,341	86,142,911	Receptor-like kinase
28	*TraesCS4B02G086400*	HC	86,359,231	86,361,142	Pectin lyase-like super-family protein

Bolded genes (based on Chinese Spring genome version 1.1) are the nearest high-/low-confidence genes linked to the SNP Excalibur_c100336_106. The annotation is based on NCBI and Ensembl

The relative CDS sequences of the above-mentioned 27 genes were obtained from the reference Chinese Spring wheat genome database RefV1.1 (https://tri-ticeaetoolbox.org/wheat//). By blasting in NCBI (https://www.ncbi.nlm.nih.gov), 11 high-confidence genes had function annotations in other plant species and two had putative functions, while nine low-confidence genes had function annotations and five had putative functions (Table 4).

Among the above-mentioned genes, *TraesCS4B02G085300* is a gene coding F-box protein, reportedly related to ABA metabolism (Lim et al. 2019; Peng et al. 2012), one of the main pathways for drought tolerance. It is the nearest upstream high-confidence gene with a distance of 0.19 Mbp to the qualified SNP (Excalibur_c100336_106). *TraesCS4B02G085400,* a high-confidence gene nearest to the SNP (41,468 bp) downstream, encodes a calcineurin-like metallophosphoesterase transmembrane protein and participates in the Ca^2+^-dependent phosphorylation regulatory system, enabling plants to tolerate various environmental stresses including drought (Sun et al. 2015; Toyota et al. 2018). The low-confidence gene *TraesCS4B02G106200LC*, annotated as a similar functional annotation with gene *TraesCS4B02G085400*, is the nearest to the SNP (Excalibur_c100336_106) with a physical distance of only 570 bp downstream. The second nearest gene (low confidence), *TraesCS4B02G106300LC*, encodes phytochelatin synthase 2.

### *In silico* expression of candidate genes

**Fig. 4:**
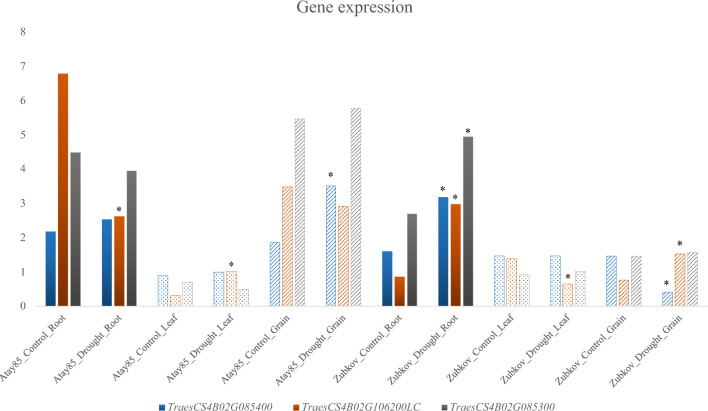
*In silico* transcriptomic expression of drought-tolerant genes. Two cultivars (Atay 85, resistant; Zubkov, susceptible) were compared under drought stress. Dots are leaf tissue, slashes are grain tissue. *Indicates a twofold or more difference in gene expression

Three candidate genes (*TraesCS4B02G106200LC*, *TraesCS4B02G085300*, and *TraesCS4B02G085400*) were further screened in WheatOmics (accessed 5 February, 2023) in gene-expression databases to analyze their expression under drought stress. The gene expression result (Fig. 4) shows that *TraesCS4B02G106200LC* expressed large variation in root and leaf tissue in drought-resistant cultivar Atay 85 (Budak 2001) and drought-susceptible cultivar Zubkov, *TraesCS4B02G085400* expressed large variation in grain tissue in the drought-resistant and susceptible cultivars, while *TraesCS4B02G085300* only expressed large variation in root tissue in the susceptible cultivar.

## Discussion

In many parts of the world, abiotic stresses such as drought have become a major factor limiting wheat production due to climate change (De Vita and Taranto 2019; Qiao et al. 2022; Zhang et al. 2022). Thus, farmers need new wheat varieties with good adaptation to the future climate. Furthermore, with the increasing shortage of water resources, there is an urgent need to breed new wheat cultivars with improved drought tolerance. Marker-assisted selection is an effective way to accelerate the breeding process and improve breeding efficiency.

This study takes advantage of newly developed NILs and the Illumina 90 K SNP chip in wheat to identify an SNP (Excalibur_c100336_106) with 100% genotype–phenotype associations among all four NIL pairs (targeting a major chromosome 4BS QTL *qDSI.4B.1* for drought tolerance) and their parents (C306 and Dharwar Dry). The SNP was converted into a KASP marker, with a confirmed 81.8% accuracy rate for evaluating drought tolerance in wheat germplasm. Consequently, the KASP marker, DE106, is a suitable molecular marker for the four tested NIL pairs and their parents and could be a highly useful molecular marker for identifying drought tolerance in practical germplasm evaluation and drought tolerance breeding of wheat.

Drought tolerance is a complicated quantitative trait, with major QTL for drought tolerance explaining limited phenotypic variation-a challenge for developing high-efficiency markers for drought tolerance (Khalid et al. 2019). Recently developed Kompetitive Allele-Specific PCR (KASP) markers are desirable for marker-assisted breeding due to their cost-effectiveness and high throughput (Han et al. 2020; Liu et al. 2015, 2022; Makhoul et al. 2020). However, very few efficient KASP markers have been reported for drought tolerance (Hao et al. 2017; Ur Rehman et al. 2021). In this study, we developed a novel KASP marker (DE106) for wheat drought tolerance using the SNP Excalibur_c100336_106 identified from four NIL pairs targeting a major QTL *qDSI.4B.1* that explained up to 14% of the phenotypic variation of drought tolerance. The KASP marker had 100% accuracy for the drought tolerance phenotype in the four tested NIL pairs and their two parents and up to 77.8% when considering 34 commercial cultivars and as high as 81.8% for the total 44 tested wheat lines collected from Australia and China with known drought tolerance/susceptibility. There are three possible reasons why the accuracy is 77.8% rather than 100%. First, drought in wheat is controlled by multiple genes, some cultivars which show drought-tolerant/susceptible phenotype may not be controlled by the QTL we investigated. Second, drought tolerance involves different metabolism pathways, not all the drought-tolerant wheats are regulated by the same pathway. Finally, some other major QTL on other chromosomes may exist which could affect the accuracy of this single QTL validation. Of course, the genetic/physical distance between the marker and the genes controlling drought tolerance/susceptibility may be the major reason. The linkage between the marker and the genes may disassociate through recombination in some genotypes resulting in lower accuracy. However, this KASP marker, associated with one of the major drought tolerance loci, could still be useful for high-throughput evaluation and marker-assisted selection of the drought tolerance trait.

**Fig. 5:**
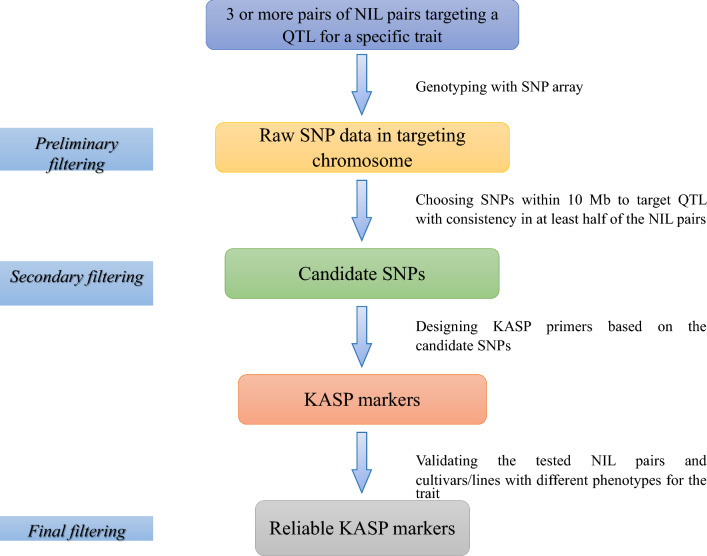
General protocol for the SQV-based approach for KASP marker development. The integrated approach uses SNP array (S), NILs targeting a QTL for a specific trait (Q), and validation using commercial varieties with differences in the specific trait (V)

According to the present work and our previous research on pre-harvest sprouting in wheat (Liu et al. 2022), integrating an SNP array, NILs targeting a QTL for a specific trait, and validation using commercial varieties with differences in the specific trait—named SQV (S = SNP, Q = QTL and V = Validation)—is a quick and efficient approach for developing KASP markers and identifying candidate genes for quantitative traits (Fig. 5). The SQV-based approach has multiple advantages. First, it could dramatically reduce researcher workload by using more than three NIL pairs with known and validated phenotypes (most tolerant/most susceptible) when screening the genetic marker data at the initial data filtering stage based on genome-wide association studies, genome re-sequencing, and/or other related methods such as DNA arrays. This study selected six candidate SNPs from the first screening by blasting the four NIL pairs targeting a QTL for drought tolerance. Other studies have also reported that using NILs as material could easily identify around 30 candidate SNPs near the target QTL region in wheat for pre-harvest sprouting resistance, heat tolerance, plant height, and root traits (Halder et al. 2021; Liu et al. 2022; Lu et al. 2020; Zhang et al. 2021), reducing the huge workload of SNP screening in wheat. Second, using target QTL makes it much easier for scientists to narrow the number of candidate SNPs. In this study, five SNPs were filtered from six after physically positioning the target QTL *qDSI.4B.1* in the reference genome. The excluded five SNPs were converted to KASP markers for further screening to verify the importance of the targeted QTL localization; the results were consistent with the prediction that these markers are inconsistent, less informative, and hence, could not be used. Third, validation with commercial cultivars could further check the broad spectrum and effectiveness of the KASP markers, an important step in our earlier study validating KASP markers developed for pre-harvest sprouting resistance (Liu et al. 2022), which narrowed ten KASP markers to four for final use. The layer-by-layer screening in the SQV approach can quickly obtain KASP markers with end-use potential (Fig. 4). Besides, this method can be used downstream in conjunction with gene mining, molecular breeding, phenotype identification, and resource variety evaluation.

The SQV-based approach should help researchers select cultivars with biotic and abiotic stress tolerance more efficiently by quickly screening and validating molecular markers and putative genes. Overall, this approach could be a simple model for developing KASP markers for quantitative wheat traits.

Drought tolerance is controlled by a number of genes and external factors. However, most external factors (e.g., light, temperature, rainfall) are hard to control in the field. Selection based on molecular markers related to drought resistance is more user-friendly and reliable for breeders. We identified a candidate gene near the SNP Excalibur_c100336_106 that generated the highly efficient KASP marker (DE106) for drought tolerance. The gene *TraesCS4B02G085300*, within 0.19 Mb of the SNP, encodes the F-box domain related to the ABA system involved in drought tolerance regulation. The F-box domain-containing protein family is one of the two largest gene families in the plant kingdom. Proteins encoded by F-box genes respond to plant abiotic stresses (Abd-Hamid et al. 2020). Among the F-box gene family, FOA1 is an ABA signaling-related gene (Peng et al. 2012). Some F-box genes, together with their interacting partners, can negatively regulate the ABA-dependent defense signaling response (Lim et al. 2019). So, *TraesCS4B02G085300* might be highly correlated with drought tolerance. *In silico* gene expression on WheatOmics revealed that *TraesCS4B02G085300* had doubled the gene expression in root tissue under drought stress compared with control, indicating that it participates in the drought response system. In addition, *TraesCS4B02G085400* (closest to the SNP with only 0.04 Mb in distance) and *TraesCS4B02G106200LC*, both encoding calcineurin-like metallophosphoesterase transmembrane protein, are ser/thr protein kinases with vital roles in plant growth, development, and multiple stress responses (Zhang et al. 2017). When calcineurin-like metallophosphoesterase transmembrane proteins interact with protein kinase TaCIPK27, *Arabidopsis* plants expressed drought tolerance and exogenous ABA sensitivity (Wang et al. 2018b). Besides, Calcineurin B-like proteins (CBLs) together with CBL-interacting protein kinases (CIPKs) are an important regulatory network (CBL-CIPK) in response to abiotic stresses (Toyota et al. 2018). The CBL-CIPK signaling system regulates many ion transport proteins. At the same time, CBL-CIPK plays an important role in the response to C/N nutrients and uptake of Mg and Fe. This Ca^2+^-dependent phosphorylation regulatory system functions to ensure plant growth and tolerate various environmental stresses, including drought (Léran et al. 2015; Ragel et al. 2019; Saito and Uozumi 2020; Sanyal et al. 2020; Zhu 2016). Another study found that calcineurin-like metallophosphoesterase transmembrane protein controls seed-coat impermeability in wild soybean (Sun et al. 2015). Hence, it might also be involved in regulating drought tolerance in wheat. The low-confidence gene *TraesCS4B02G106200LC* is the nearest one to the SNP (Excalibur_c100336_106) with a physical distance of only 570 bp downstream, with similar functional annotation as the gene *TraesCS4B02G085400*. These two genes may be adjacent genes that co-regulate the drought response. *In silico* gene expression on WheatOmics showed that *TraesCS4B02G085400* and *TraesCS4B02G106200LC* expressed two-fold or more differences in gene expression in root and grain tissue under drought stress, indicating that they also participate in the drought response system. Consequently, our study improves our understanding of the genes and genetic pathways controlling drought tolerance in wheat.

## Conclusion

We identified a novel SNP, Excalibur_c100336_106, with 100% consistency of genotype–phenotype associations among four NIL pairs targeting a major 4BS QTL (*QDSI.4B.1*) responsible for drought tolerance and their two parents in wheat. A highly efficient KASP marker generated from this SNP had 81.8% conformity of the drought-tolerant genotype and phenotype when tested in 30 commercial cultivars with known drought responses, the four NIL pairs, and their parents. The SNP-converted KASP marker can be suitably employed in future genetic studies on drought tolerance and for high-throughput germplasm evaluation and marker-assisted breeding in wheat. Two candidate genes were found near this SNP—*TraesCS4B02G085400* (0.04 Mb) encoding a calcineurin-like metallophosphoesterase transmembrane protein and *TraesCS4B02G085300* (0.19 Mb) encoding an F-box protein—which might be responsible for drought tolerance in wheat.

## Supplementary Information

Below is the link to the electronic supplementary material.

Supplementary file1 (XLSX 18 KB)

## Data Availability

The data supporting this study’s findings are available in the supplementary material.
